# Peptide-Protein Interaction Studies of Antimicrobial Peptides Targeting Middle East Respiratory Syndrome Coronavirus Spike Protein: An In Silico Approach

**DOI:** 10.1155/2019/6815105

**Published:** 2019-07-01

**Authors:** Sabeena Mustafa, Hanan Balkhy, Musa Gabere

**Affiliations:** ^1^Department of Biostatistics and Bioinformatics, King Abdullah International Medical Research Center, King Saud bin Abdulaziz University for Health Sciences, Riyadh, Saudi Arabia; ^2^Infection Prevention and Control Department at the Ministry of National Guard, Department of Infectious Diseases, King Abdullah International Medical Research Center, King Saud bin Abdulaziz University for Health Sciences, Riyadh, Saudi Arabia

## Abstract

There is no effective therapeutic or vaccine for Middle East Respiratory Syndrome and this study attempts to find therapy using peptide by establishing a basis for the peptide-protein interactions through in silico docking studies for the spike protein of MERS-CoV. The antimicrobial peptides (AMPs) were retrieved from the antimicrobial peptide database (APD3) and shortlisted based on certain important physicochemical properties. The binding mode of the shortlisted peptides was measured based on the number of clusters which forms in a protein-peptide docking using Piper. As a result, we identified a list of putative AMPs which binds to the spike protein of MERS-CoV, which may be crucial in providing the inhibitory action. It is observed that seven putative peptides have good binding score based on cluster size cutoff of 208. We conclude that seven peptides, namely, AP00225, AP00180, AP00549, AP00744, AP00729, AP00764, and AP00223, could possibly have binding with the active site of the MERS-CoV spike protein. These seven AMPs could serve as a therapeutic option for MERS and enhance its treatment outcome.

## 1. Introduction

Middle East Respiratory Syndrome-Coronavirus (MERS-CoV) was identified in Saudi Arabia in 2012 and it belongs to Coronaviridae family and mostly reported among the Middle Eastern people. This virus causes the respiratory illness called the Middle East Respiratory Syndrome (MERS) [1]. Phylogenetic studies show that bats are the reservoir of this virus and camel is the only host through which the virus spreads to humans [2].

According to the World Health Organization (WHO), at the end of November 2018, a total of 2274 laboratory-confirmed cases of Middle East Respiratory Syndrome (MERS), including 806 associated deaths, were reported globally, where the majority of these cases were reported from Saudi Arabia (1896 cases, including 732 related deaths). Although different classes of treatment trials are ongoing, no effective treatment or vaccine is available for this disease, which causes the necessity of the effective therapeutic treatments. In this scenario, peptides can serve as potential treatment option for MERS. It has been shown that peptides act as modulators in viral diseases. For example, Melnik et al. [3] shortlisted nine peptides based on the Wimley-White interfacial hydrophobicity scale (WWIHS), where four of these peptides (WWIHS = 3.5) had greater than 50% inhibition of human cytomegalovirus. In another study, it was shown that several peptides with WWIHS = 5.2 inhibited multiple strains of influenza with IC_50_≤1*μ*M [4]. By and large, other peptides with positive WWIHS values have been shown to inhibit various viruses such as Rift Valley Fever [5], Dengue, White Nile [6], and SARS [7] and the mechanism of action is by interfering with fusion of host cellular and viral glycoprotein membranes [8]. It is for these reasons we propose that antimicrobial peptides (AMPs) can be used as an effective therapeutic agents against MERS. Several peptides have been extensively studied and identified as anti-MERS-CoV peptides [9–12] and anti-MERS-CoV AMPs in the past few years [13].

In order to target MERS-CoV, the knowledge of structural and nonstructural proteins is important. In this work, we focus on the structural protein of MERS-CoV spike (S) protein. Understanding the S protein structure is useful in the drug discovery for developing anti-MERS-CoV components. The S protein consists of S1 and S2 regions and it is a type I transmembrane glycoprotein, which is located at the viral envelope surface in a trimer state. The S1 and S2 subunits play a role in viral entry, binding, and fusion [14]. The S1 subunit has a receptor-binding domain (RBD) which binds to the receptor dipeptidyl peptidase 4 (DPP4). The S2 subunit consists of heptad repeats 1 and 2 (HR1 and HR2), which forms a complex called the fusion core, and represents a key membrane fusion architecture [14]. During the process of membrane fusion, HR1 and HR2 regions form a six helix bundle core with a hydrophobic region being inserted into the host membrane and thereby fusion occurs [15].

Proteins interact with other proteins in order to perform cellular tasks and knowledge of this can facilitate the development of therapeutics. X-ray crystallography and mutagenesis are techniques used in determining protein complexes and consequently protein interfaces. However, these techniques are expensive; hence, an in silico approach in predicting protein interaction, protein-protein docking, and protein interface is needed. Protein-peptide docking methods can be divided into three categories: template-based docking; local docking; and global docking [16]. Example of protein-protein interactions includes the use of graphical models by predicting the binding site between two proteins [17]. Protein interface prediction involves determining a subset of residues on the protein surface which are involved in intermolecular interactions. Example of prediction of protein interfaces includes ComplexContact, which is a web server for determining interfacial residue-residue contact prediction of a putative protein complex. This is useful in deciphering how proteins form a complex by looking at how their residues interact [18]. The challenges faced in developing these computational methods are that no methods yield excellent results and there is no gold standard benchmark dataset that can be used to compare them [19]. In addition, there are other challenges like (i) modelling significant conformational changes of both peptide and protein molecules, (ii) selection of the highest accuracy structure out of many generated models, and (iii) integration of experimental data and computational predictions into the protein-peptide docking scheme [16].

In this study, we have considered a set of AMPs in order to identify their role as putative modulators for MERS-CoV proteins. More specifically, we aim at evaluating the inhibitory mechanism of a set of AMPs with specific physicochemical properties and by employing peptide-protein interaction in order to determine its accuracy in binding with spike fusion core of MERS-CoV. The motivation for this study is that we will use highly available and reusable data that would otherwise be costly to produce. In addition, the simplicity of the proposed method, once optimized, will make it easy to identify the most important peptides that act as therapeutic agents. Our study is purely based on the binding efficacy of antimicrobial peptides on MERS-CoV spike (S). The consequences of such effort are twofold: (i) it will eliminate or at the least minimize the cost of synthesizing countless numbers of peptides and (ii) accelerate drug discovery of MERS therapeutics.

## 2. Materials and Methods

**Figure 1:**
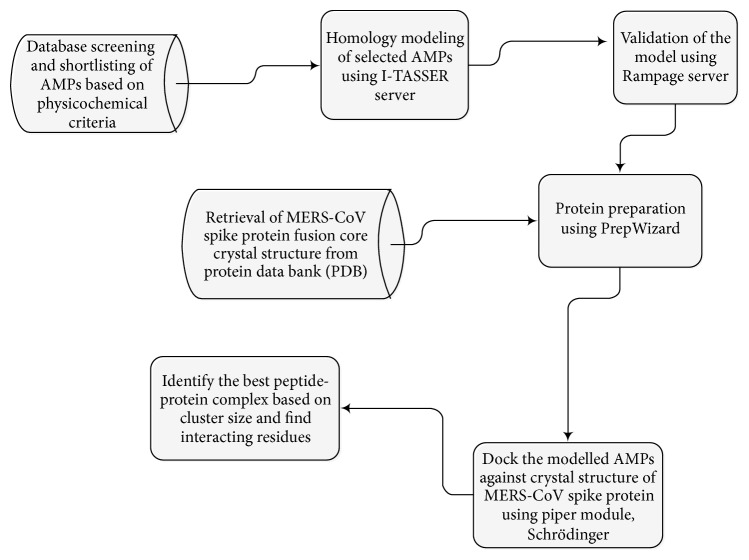
The flowchart depicting the methodology employed in this study.

We propose a two-stage computational approach to determine possible antimicrobial peptides (AMPs) that can target spike protein of MERS-CoV. The first stage involves database screening of AMPs from APD3 database (http://aps.unmc.edu/AP/main.php) [15] based on physicochemical properties. The second stage involves the structural bioinformatics studies to analyze the peptide-protein interaction complex of MERS-CoV S protein using the shortlisted AMPs and implement the docking studies in order to determine the interacting residues with greater affinity. The flowchart depicting the methodology employed in this study is shown in Figure 1. Detailed description of the steps involved is presented in the following subsections.

### 2.1. Retrieval of the Prefusion Structure of MERS-CoV spike Protein

**Figure 2:**
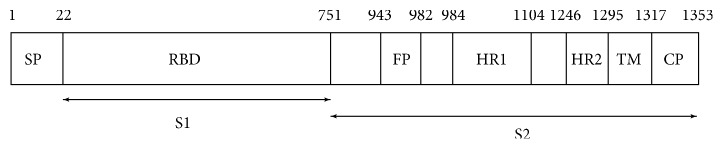
MERS-CoV spike (S) protein and its S2 regions which form a fusion core HR1 and HR2 are the heptad repeats 1 and 2 [39].

**Figure 3:**
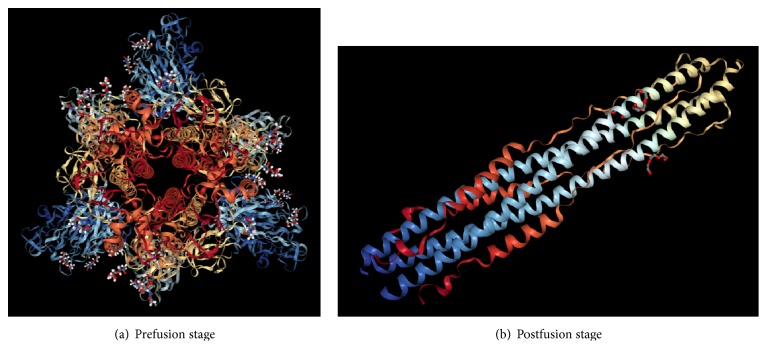
(a) Prefusion stage (PDB ID: 5X59) of the S protein and (b) S2 protein forms a six-helical bundle (6-HB) during postfusion stage (PDB ID: 4NJL.

We considered the S protein of MERS-CoV, which is illustrated in Figure 2; in particular, we selected the prefusion form. The rationale for targeting HR regions in the prefusion conformation is because the antimicrobial peptide can potentially prevent protein refolding and fusion. Hence, this will prevent the formation of the 6-HB (postfusion) and the entry of the virus into the host cell. It is for this reason we retrieved the cryo-EM structure of the MERS S spike protein from Protein Data Bank (PDB) with the PDB ID: 5X59 [20], which is a prefusion structure of MERS-CoV spike glycoprotein with threefold symmetry as shown in Figure 3(a). The postfusion of MERS-CoV spike protein is shown in Figure 3(b). The structure was elucidated by electron microscopy with a resolution of 3.7Å. This structure has total weight of 444204.84 and a sequence length of 3969 amino acid residues.

### 2.2. Database Screening of Antimicrobial Peptides

The set of AMPs were retrieved from the antimicrobial peptide database (version 3), APD3 [15]. This database contains a total of 2961 AMPs from six kingdoms, namely, bacteria, archaea, protists, fungi, plants, and animals. In particular, we selected a list of basic antimicrobial peptides that are broad-spectrum. The strategy employed is based on a similar database screening [21] with additional criteria. The extracted AMPs from APD3 were filtered according to the following criteria, namely,

- 20aa < = length < = 55aa: The longer the sequence, the better the antiviral activity [14],
- basic residues should be abundant [13],
- net charge > = 0 because the virus membrane is negatively charged [21],
- nontoxic to mammalian cells [21],
- peptides with unknown anti-MERS-CoV activity,
- not annotated as synthetic (i.e., man-made peptides) in the database [21],
- interfacial activity [8] should be as follows:
  - Wimley-White interfacial hydrophobicity scale (WWIHS > 0),
  - interfacial helical hydrophobic moment (iHHM > 0).

In addition to the list of AMPs, we selected a number of peptides from the literature, which have been verified experimentally to have anti-MERS-CoV activity. These peptides act as positive control in which their docking complexes with MERS-CoV will be compared with our predicted complexes, in particular, two positive controls, namely P9 [13] and HR2P [10]. P9 is a subsequence derived from mouse beta defensin (mBD4) while HR2P is a peptide from the HR2 region of MERS-CoV spike protein.

### 2.3. Ab Initio Modelling of the Shortlisted AMPs and Validation

The 3D structure of the shortlisted AMPs was predicted by submitting amino acid sequence into I-TASSER (https://zhanglab.ccmb.med.umich.edu/I-TASSER/), an online server which stands for Iterative Threading ASSembly Refinement [22]. This integrated platform works based on sequence-structure-function relation. This is an automated modelling server, which predicts a model based on confidence score (C-score) and build five models with confidence ranging from -5 to 2. C-score gives the estimate of accuracy of the prediction. If the C-score increases, the confidence of the model also increases. Based on C-score, best model of the peptide was selected for the study. The predicted AMPs structures obtained from I-TASSER were subjected for validation using Rampage server (http://mordred.bioc.cam.ac.uk/~rapper/rampage.php) [23]. This validation recognizes errors in theoretical models of protein structures by performing statistical analysis of all available protein structures.

### 2.4. Protein Preparation of the Receptor (5X59) and the Ligand AMPs

The protein preparation wizard (PrepWizard) was used to prepare the structure of the modelled AMPs and also the crystal structure of the fusion core spike protein S2 (5X59). The aim of protein preparation was to optimize the molecule (Schrödinger Suite 2018 Protein Preparation Wizard, Schrödinger LLC, New York, NY, 2018). The protein chains were edited for missing hydrogen atoms; bond orders and hydrogen bonds were optimized. The preparation process of the protein continued until it attained a minimized state, which usually has a default Root Mean Square Deviation (RMSD) value of 0.30.

### 2.5. AMPs-Receptor Docking

To determine the binding mode of shortlisted AMPs with the spike protein receptor (5X59), 5X59 and the modelled AMPs were subjected to peptide-protein docking using Piper module of Schrödinger [24, 25]. Piper algorithm is based on fast Fourier transform and it consists of two steps, namely, conformational sampling and structural clustering. The conformational sampling involves performing exhaustive evaluation of an energy function given in Equation (1) [25] in a discretized space of mutual orientations of two proteins. On the other hand, the structural clustering aids in identifying and ranking likely docked protein poses.

(1) $$\begin{array}{c} E\left(\;\alpha ,\beta ,\gamma \;\right)=\underset{p}{\sum }\;\underset{i,j,k}{\sum }\left(\;R_{p}\left(\;i,j,k\;\right)L_{p}\left(\;i+\alpha ,j+\beta ,k+\gamma \;\right)\;\right) \end{array}$$

For peptide-protein docking, the AMPs were set as ligands and docked with receptor 5X59. The number of ligand rotation to probe was set for 10,000 rotations and, for each dock, five poses were retrieved. This was done in order to find large clusters of structures below a certain energy value. The shortlisted AMPs with the best Piper cluster size than experimentally validated peptides against MERS-CoV are considered as putative anti-MERS-CoV AMPs. In addition, we have used ClusPro 2.0 server [26] in determining protein-protein interaction. Briefly, ClusPro, rotates the ligand with 70,000 rotations and, for each rotation, it translates the ligand in *x*, *y*, and *z* axis relative to the receptor on a grid. The ClusPro 2.0 server is based on Piper, but the method is extended to be used with pairwise interaction potentials [26].

### 2.6. Binding Mode of Docked Complexes

The docked complex structure output format was submitted into the Protein Interactions Calculator (PIC) webserver (http://pic.mbu.iisc.ernet.in/) in order to map the interaction of the resulting docked complex [27]. The parameters such as number of hydrogen bonds, number of hydrophobic residues, and number of aromatic and ionic interactions were considered in interpreting the strength of the interaction.

## 3. Results

### 3.1. Peptide Modelling Using I-TASSER and Validation

As we theorize that the spike protein of MERS-CoV represents the key receptor for our analysis, we focus on developing a theoretical model for the selected AMPs using I-TASSER server and its evaluation using Ramachandran plot. The Ramachandran plots of the theoretical models were developed and compared using a server, namely, Rampage. Out of the 37 models developed, most of the models had good quality score and backbone conformation which are considered as reliable. Supplementary Table 1 represents the number of residues in the favored region, allowed region, and outlier region in 13 models predicted.

### 3.2. Filtering of AMPs Based on Database Screening Criteria

The filtering process using the criteria mentioned in Section 2.2 resulted in 37 shortlisted AMPs as shown in Table 1, where majority of the AMPs belong to the defensin family from different species.

### 3.3. Protein Preparation, Docking, and Evaluation of Top Complexes

The PrepWizard prepared the structures by automatically adding missing hydrogen atoms and correcting bond order assignments, charge states, and orientation of various groups and performed restrained minimizations which allow hydrogen atoms to be freely minimized.

Further, AMP-MERS docking (docking of antimicrobial peptides with 5X59) was performed by using Piper algorithm and the pose with the best fit was selected for each peptide-protein complex based on cluster size. The resulting structures were grouped into clusters and ranked according to cluster size values from the largest to the smallest. Top ranked peptide and protein complex details are presented in Tables 2, 3, and 4.

The results indicate that, out of 37 AMPs, 8 AMPs had a cluster size greater than 200 which is an indication of the binding of the peptides to MERS-CoV spike protein as shown in Table 2. These AMPs are derived from various sources such as fungus, plants, and fish. In addition, it was found that the seven AMPs had higher cluster size value than the positive control (HR2P: cluster value of 208), but lower cluster size value than the positive control (P9: cluster value of 328), where HR2P and P9 have been experimentally demonstrated to possess potent anti-MERS-CoV activity [10, 13]. In particular, we considered a cutoff value of 208. The positive control P9 was the best and had a cluster size value of 328 as shown in Table 2. Peptide AP00225 showed a very strong binding affinity score with a cluster size (binding affinity score) of 285 compared to all other putative peptides. Other top ranked putative AMPs include AP00180, AP00549, AP00744, AP00729, AP00764, and AP00223 with cluster size values of 277, 270, 253, 247, 223, and 219, respectively. These confirm the probability of these five peptides to be putative anti-MERS-CoV peptides. For further analyses, we selected four peptides, namely, AP00225, AP00180, AP00549, and AP00744, belonging to family of defensin. During validation of these four putative defensin peptides, AP00225 Ramachandran plot (Psi-Phi) pairs had 79.3% of residues in most favored regions, 6.9% core residues in allowed regions, and 13.8% residues in outlier regions. AP00180 Ramachandran plot (Psi-Phi) pairs had 84.3% of residues in most favored regions, 10.0% core residues in allowed regions, and 6.7% residues in outlier regions. AP00549 Ramachandran plot (Psi-Phi) pairs had 81.6% of residues in most favored regions, 7.9% core residues in allowed regions, and 10.5% residues in outlier regions. AP00744 Ramachandran plot (Psi-Phi) pairs had 87.2% of residues in most favored regions, 7.7% core residues in allowed regions, and 5.1% residues in outlier regions (Supplementary Table 1).

In addition, the results show that 20 AMPs and 12 AMPs were ranked higher than P9 and HR2P, respectively, shown in Table 3. However, the ranking based on energy scores shows that 11 AMPs and 17 AMPs were ranked higher than HR2P and P9 as tabulated in Table 4. We have used the results given by Piper (Table 2) and have used cluster size because, in ClusPro 2.0 documentation, they mentioned that the best way to rank models is by cluster size and not by energy scores.

### 3.4. Evaluation of Peptide-Protein Complex and Its Interactions Analysis

**Figure 4:**
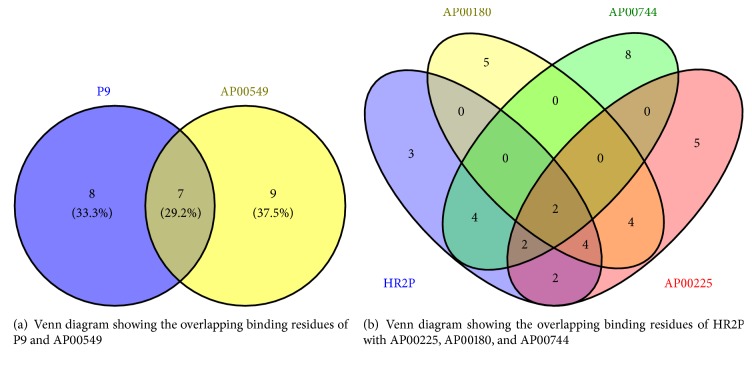
Venn diagram showing common residues.

**Figure 5:**
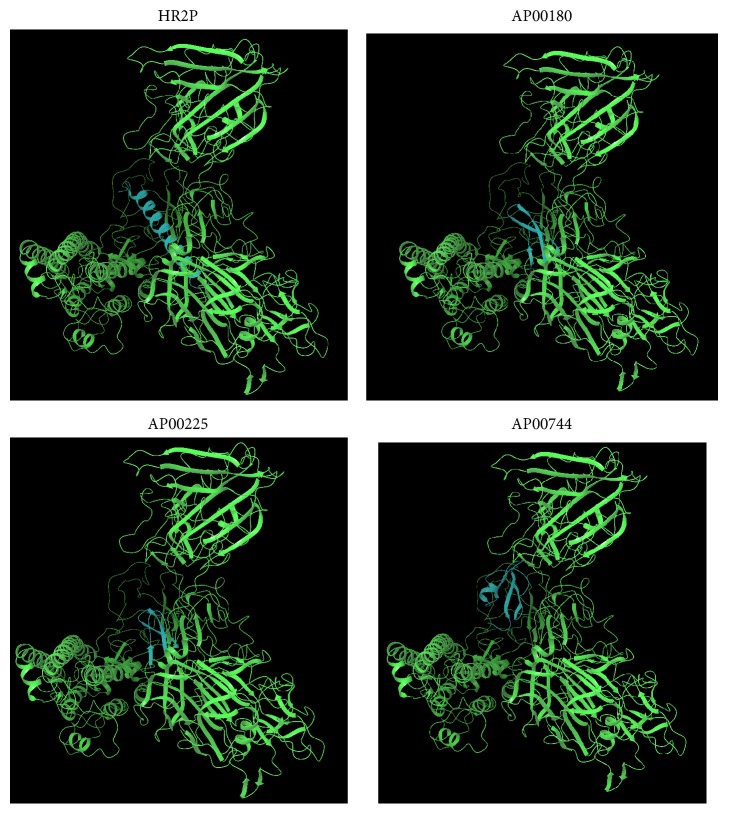
Possible interaction of receptor spike and ligands, namely, HR2P, AP00180, AP00225, and AP00744, which all bind to the spike protein in similar position.

**Figure 6:**
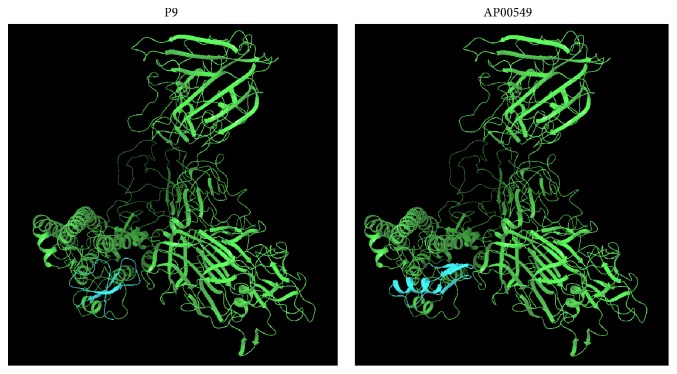
Possible interaction of receptor spike and ligands, namely, P9 and AP00549, which all bind to the spike protein in similar position.

**Figure 7:**
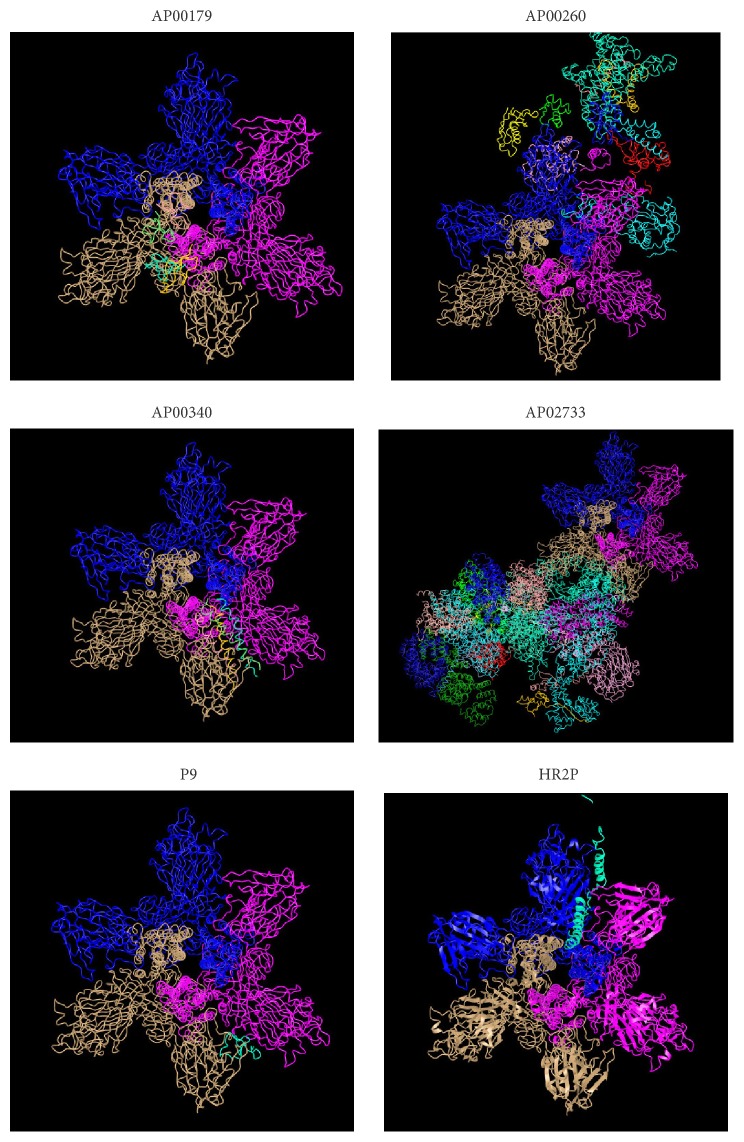
Possible interaction of receptor spike and ligands, namely, AP00179, AP00260, AP00340, AP02733, P9, and HR2P.

Once we observed that the AMPs could potentially bind to spike protein, the next step was to know the binding mode. In particular, we have used Protein Interactions Calculator (PIC) to recognize the interactions within the bound complexes. In structural bioinformatics, predicting protein-protein interactions which stabilize the tertiary and quaternary structures is an important task. For the top best four AMPs-MERS-CoV complexes with the best cluster size were subjected to PIC server and the binding mode (interactions) of each peptide are given in Table 5. PIC identified interactions such as hydrophobic residues interactions, ionic interactions, hydrogen bonds, aromatic-aromatic interactions and aromatic–sulphur interactions within the peptide-protein complexes. According to the PIC server results as shown in Table 5, AP00225 forms hydrophobic interactions with Val790, Tyr1142, Phe764, Leu731, Ile768, Pro1143, Pro767, and Val770; hydrogen bond interactions with Pro730; and ionic interactions with Gln792 and Ser734 as shown in Table 5. AP00180 forms hydrophobic interactions with Ala1007, Val790, Leu731, Pro767, Ile768, and Tyr1142; hydrogen bond interactions with Gly789 and Pro730; and ionic interactions with Glu1017 and Asp740 as shown in Table 5. AP00549 forms hydrophobic interactions with Ala1049, Pro59, Tyr64, Tyr928, Val929, Ala930, Ala920, Ile69, and Tyr71; hydrogen bond interactions with Ala1049 and Gly61; and ionic interactions with Arg1057, Arg62, and Asp922 as shown in Table 5. AP00744 forms hydrophobic interactions with Leu1200, Pro767, Val1168, Ile1180, Leu780, Phe778, Pro1143, Val983, and Ile985; hydrogen bond interactions with Ala1206; and ionic interactions with Asp771 as shown in Table 5. These residues may be considered as critical residues. AP00549 have overlapping residues with experimentally validated anti-MERS-CoV peptide P9, as highlighted in bold (Table 5) and Figure 4(a), while AP00225, AP00180, and AP00744 have common residues with HR2P as highlighted in italic (Table 5) and Figure 4(b). The binding of the peptide AP00549 has the same binding region as P9; AP00225, AP00180, and AP00744 have the same binding region as shown in Figures 5 and 6. The binding of the peptides to the receptor spike and ligands includes AP00179, AP00260, AP00340, AP02733, P9, and HR2P; see Figure 7.

## 4. Discussion

Computational and structural biology methods have accelerated the discovery of novel drugs used to treat viral diseases [21, 28]. We followed the structural biology aspects which focus on the availability and retrieval of an S protein receptor structure from PDB which was resolved using cryo-EM structure method. We have applied the docking technique not only to predict the binding mode of AMPs to spike protein but also to study the peptide-protein interactions. The receptor used is the prefusion state of the S protein, because it is a type I fusion protein, which undergoes a nonreversible conformational change that results in the postfusion form of the protein. In postfusion, the protein has refolded and the membranes have undergone fusion, or the spike protein has been spent. Therefore, it is reasonable to target the S protein in the prefusion conformation as peptides bind to the prefusion conformation. This can potentially prevent protein refolding and entry of the virus into the host cell, as it has been shown for diverse Type I proteins like HIV gp41 and paramyxovirus F proteins. Once the 6HB fusion core is formed, it will not accommodate any peptide within it and any peptide related therapeutic intervention would have to take place either at the prefusion stage, or at the intermediate hairpin stages [29].

The model refinement has improved its quality of the theoretical models developed for selected AMPs. Further, Ramachandran plots from Rampage server determined the stereochemical quality of best scored four defensin models. The aim of model refinement using Rampage Ramachandran plot was to determine whether the theoretical model acquired the quality and side chain configuration [30]. This program helps researchers to evaluate the accuracy of the predicted models [31].

Based on filtering of potential peptides acting against S protein, we employed interfacial activity based on WWIHS and iHMM scales. It has been hypothesized that positive interfacial hydrophobicity of a peptide increases the chances for membrane binding, that is, interacting with the viral hydrophobic surfaces and hence inhibiting fusion and entry of the virus [8]. Therefore, since the best five putative AMPs have higher values of interfacial hydrophobicity, we can infer that their mechanism of action is by inhibiting fusion and hence blocking entry of the virus into the host cell. The mechanism of action for the positive controls, namely, P9 [13] and HR2P [10] has been experimentally validated to inhibit viral fusion. Nevertheless, these five putative anti-MERS-CoV AMPs have same values as positive control peptides in terms of WWHIS and iHMM values and also on an average sequence length.

In order to characterize binding properties of AMPs with the spike protein S2, we used Piper, which involves peptide-protein interaction, in which it determines the best-fit orientation of ligand with receptor [25]. The binding affinity is determined by Piper cluster size and not scores or probability. The docking score together with probability can give confidence on the binding and which are lacking in our study since we used cluster size as criteria for ranking best poses. However, it has been hypothesized that the best way to rank docking is by cluster size, which can be useful in informing experimental approaches [25]. In this study, we selected five peptides which had higher cluster value than experimentally validated anti-MERS-CoV peptide, P9 [13]. Nevertheless, these peptides have good binding with MERS-CoV spike protein (S2) in terms of hydrogen bonds and hydrophobic interactions.

Our results may indicate that AP00225 (Rat defensin), AP00180 (Human alpha defensin), AP00549 (Plectasin), AP00744 (Chicken beta defensin), AP00729 (Cyclotides), AP00764 (Dermaseptin-S9), and AP00223 (Rat alpha defensin) were the best docked peptides with high cluster size values, which is an indicative of strong binding affinity. Most of these putative peptides are defensin, similar to P9, which is a mouse beta defensin. On comparison of P9 and AP00549, we found that five residues, namely, Tyr64, Ile69, Ala920, Tyr928, and Val929, were common in the binding. On the same breath, HR2P have nine common residues (Leu780, Pro767, Tyr1142, Leu729, Leu731, Val1168, Val770, Pro1143, Val790) with AP00225, AP00180, and AP00744. These residues may be considered as key or critical and may play a major role in the protein protein-interaction [32] and might inhibit the formation of the six-helical bundle (6-HB). Further studies may help to understand the role of these residues in drug binding mechanism.

Interestingly, these seven best peptides have been experimentally validated to have activity against various microorganisms. For instance, AP00225 and AP00223 are rat defensin and this peptide has activity against* Escherichia coli* ML-35,* Acinetobacter calcoaceticus* HON-1,* Staphylococcus aureus* 502A, and* Candida albicans* 820 in vitro [33]. AP00180 is a Human alpha defensin and it inhibits nonenveloped BK virus infection [34]. AP00549 is fungal plectasin which belongs to the family of defensins and primary source is* Pseudoplectania nigrella* and this peptide is an effective antiviral against dengue virus [31]. AP00744 (Chicken avian beta defensin) has antimicrobial activity against* Salmonella serovars* [35]. AP00729 is kalata B1 which is generally known as plant cyclotides acting as a stable component for drug discovery [36]. Cyclotides possess many biological activities such as anti-HIV, antimicrobial, and hemolytic. Some cyclotides show cell penetrating properties. AP00764 (dermaseptin) induces the migration of immune cells. Dermaseptin shows inhibition against the microorganisms and cancer cell lines [37]. This peptide has innate immunity properties [38]; however, it has not been shown to have antiviral activity against MERS-CoV. This could suggest that mechanism of action of this and other peptides could be varied.

Finally, lack of wet-lab validation is a drawback in our research and we expect computational biology analysis and its integration with wet-lab data can be productive in the determination of potential anti-MERS-CoV components.

## 5. Conclusion

In the present work, we have used a docking and scoring algorithm for combined peptide-protein binding mode search using a list of AMPs. We named the method as AMP-MERS docking as it is a novel application of AMPs to MERS-CoV spike protein. Our computational study confirms that four AMPs were able to bind clearly to the specific binding site of S protein (5X59). From our results, it may confirm that these AMPs may be suitable for inhibiting MERS-CoV virus entry into the host cell by binding and preventing fusion. However, the results are preliminary and certainly need experimental confirmation using in vitro and in vivo experiments essential to validate them. Special assays studies are needed to confirm the mechanism of action. Considering all the structural aspects and binding affinity studies of the four AMPs may possibly be the first choice as an anti-MERS-CoV AMPs which could be exploited to design potential inhibitors for treating MERS. We conclude that molecular docking studies aid in deciphering the antiviral activity of molecules by determining the inhibition score and binding energy.

## Figures and Tables

**Table 1:** The table shows the filtering of antimicrobial peptides from various sources based on length, WWIHS, iHMM, and net charge.

S.No	Peptide	APD3 ID	Definition	Length	WWIHS	iHHM	Net charge
1	KTCENLADTFRGPCFATSNC	AP00532	Lunatusin	20	1.8	2.62	0
2	GLFVGVLAKVAAHVVPAIAEHF	AP00260	Maculatin 1.1	22	1.29	3.26	1
3	GIGKFLHSAGKFGKAFVGEIMKS	AP00771	Magainin 1	23	1.34	7.19	3
4	GIGKFLHSAKKFGKAFVGEIMNS	AP00144	Magainin 2	23	0.93	7.1	3
5	GEGFLGMLLHGVGHAIHGLIHGK	AP02663	Piscidins	23	1.26	4.72	0
6	GLRSKIWLWVLLMIWQESNKFKKM	AP00764	Dermaseptin-S9	24	6.07	2.95	4
7	FLPVLAGIAAKVVPALFCKITKKC	AP00074	Brevinin-1	24	1.31	3.48	4
8	GWGSFFKKAAHVGKHVGKAALTHYL	AP00166	Pleurocidin	25	0.37	7.49	4
9	FFGWLIRGAIHAGKAIHGLIHRRRH	AP00340	Chrysophsin-2	25	0.02	5.97	0
10	ALWMTLLKKVLKAAAKAALNAVLVGANA	AP00160	Dermaseptin-S4	28	0.92	4.71	4
11	GLPVCGETCVGGTCNTPGCTCSWPVCTRN	AP00729	Kalata B1	29	1.94	3.64	0
12	GAFGNFLKGVAKKAGLKILSIAQCKLSGTC	AP01644	Brevinin-2-RN1	30	0.62	1.97	5
13	GWFKKAWRKVKNAGRRVLKGVGIHYGVGLI	AP00692	Hagfish cathelicidin	30	0.24	6.41	8
14	GSVLNCGETCLLGTCYTTGCTCNKYRVCTKD	AP00730	Kalata B8	31	2.47	2.06	1
15	RRCICTTRTCRFPYRRLGTCIFQNRVYTFCC	AP00174	Guinea pig neutrophil	31	2.16	2.06	7
16	ACYCRIGACVSGERLTGACGLNGRIYRLCCR	AP00225	RatNP-4 rat defensin,	31	2.43	2.58	4
17	GVIPCGESCVFIPCISTLLGCSCKNKVCYRN	AP00275	Circulin B	31	2.44	3.23	2
18	GVIPCGESCVFIPCISAAIGCSCKNKVCYRN	AP01022	Cycloviolin A	31	1.43	2.52	2
19	KIPCGESCVWIPCVTSIFNCKCKENKVCYHD	AP01061	Circulin D	31	1.27	5.43	1
20	GSIPACGESCFKGKCYTPGCSCSKYPLCAKN	AP01065	Cycloviolacin O14	31	0.73	1.48	3
21	CGESCVFIPCITTVLGCSCSIKVCYKNGSIP	AP02571	Cycloviolacin VY1	31	3.15	2.77	1
22	ATCYCRTGRCATRESLSGVCEISGRLYRLCCR	AP00180	Human defensin 5	32	1.39	3.83	4
23	AFTCHCRRSCYSTEYSYGTCTVMGINHRFCCL	AP00181	Human defensin 6	32	3.82	2.56	2
24	VTCYCRRTRCGFRERLSGACGYRGRIYRLCCR	AP00222	RatNP-1 rat defensin	32	1.02	4.29	8
25	VTCYCRSTRCGFRERLSGACGYRGRIYRLCCR	AP00223	RatNP-2 rat defensin	32	1.7	4.01	7
26	GFFALIPKIISSPLFKTLLSAVGSALSSSGEQE	AP00641	Pardaxin 1	33	4.19	0.2	0
27	VCSCRLVFCRRTELRVGNCLIGGVSFTYCCTRV	AP00179	Human neutrophil peptide-4	33	3.28	1.54	4
28	DFASCHTNGGICLPNRCPGHMIQIGICFRPRVKCCRSW	AP00036	Bovine beta-defensin 1	38	0.22	4.72	4
29	GFGCNGPWDEDDMQCHNHCKSIKGYKGGYCAKGGFVCKCY	AP00549	Plectasin	40	2.11	3.67	1
30	GLPQDCERRGGFCSHKSCPPGIGRIGLCSKEDFCCRSRWYS	AP00744	Chicken AvBD5	41	0.93	3.26	3
31	SPIHACRYQRGVCIPGPCRWPYYRVGSCGSGLKSCCVRNRWA	AP00742	Chicken AvBD6	42	0.58	2.64	7
32	KYYGNGVSCNKKGCSVDWGKAIGIIGNNSAANLATGGAAGWKS	AP00846	Mundticin KS	43	0.83	2.1	4
33	QEAQSVACTSYYCSKFCGSAGCSLYGCYLLHPGKICYCLHCSR	AP01788	Myticin C	43	4.62	0.95	2
34	VSFPWSCAALSGVCRQGACLPSELYFGPLGCGKGSLCCVSYFL	AP02830	Channel catfish beta defensin	43	8.81	2.3	1
35	KTCMTKKEGWGRCLIDTTCAHSCRKYGYMGGKCQGITRRCYCLLNC	AP01356	Cp-thionin II	46	0.39	2.59	7
36	FFLLFLQGAAGNSVLCRIRGGRCHVGSCHFPERHIGRCSGFQACCIRTWG	AP02148	Apl-AvBD16	50	3.26	5.26	5
37	LFGSVKAWFKGAKKGFQDYRYQKDMAKMNKRYGPNWQQRGGQEPPADAQANDQPP	AP02733	Piscidin 6	55	2.93	6.41	5

**Table 2:** Piper ranking of docked complex based on cluster size, where peptides with (*∗*) represent the experimentally validated against MERS-CoV and are considered as positive controls.

Rank	Peptide	Length	Definition	Species	Cluster
*1 *	* P9∗*	*30 *	*Mouse Beta-defensin *	* Mouse *	*328*
2	AP00225	31	RatNP-4 (rat defensin)	Rat	285
3	AP00180	32	Human defensin 5 (alpha defensin)	Human	277
4	AP00549	40	Plectasin (fungal defensin)	Fungus	270
5	AP00744	41	AvBD-5, chicken avian beta defensin)	Chicken	253
6	AP00729	29	Kalata B1 (cyclotides)	Plant	247
7	AP00764	24	Dermaseptin-S9	Frog	223
8	AP00223	32	RatNP-2 (rat alpha defensin)	Rat	219
*9*	* HR2P∗*	*36 *	*HR2 region of MERS-CoV *	*Synthetic *	*208*

10	AP00160	28	Dermaseptin-S4	Frog	200
11	AP00174	31	Guinea pig neutrophil cationic peptide 1	Guinea pig	193
12	AP00730	31	Kalata B8 (cyclotides)	Plant	175
13	AP00222	32	RatNP-1 (rat alpha defensin,)	Rat	175
14	AP02663	23	Piscidins	Fish	171
15	AP01061	31	Circulin D (cyclotides)	Plant	163
16	AP01356	46	Cp-thionin II	Plant	160
17	AP00532	20	Lunatusin	Plant	157
18	AP02571	31	Cycloviolacin VY1 (cyclotides)	Plant	155
19	AP00692	30	HFIAP-3 (Hagfish cathelicidin)	Fish	148
20	AP00260	22	Maculatin 1.1	Frog	146
21	AP02733	55	Piscidin	Fish	144
22	AP00275	31	Circulin B (cyclotides)	Plant	143
23	AP01644	30	Brevinin-2-RN1	Frog	143
24	AP02148	50	Apl-AvBD16 (Beta def)	Bird	139
25	AP01065	31	Cycloviolacin 014 (cyclotides)	Plant	136
26	AP01022	31	Cycloviolin A (cyclotides)	Plant	134
27	AP00036	38	Bovine Beta-defensin 1	Bovine	127
28	AP00074	24	Brevinin-1	Frog	120
29	AP00340	25	Chrysophsin-2	Fish	120
30	AP00166	25	Pleurocidin	Fish	118
31	AP02830	43	ccBD (Channel Catfish beta def)	Fish	115
32	AP00181	32	Human defensin 6	Human	115
33	AP01788	43	Myticin C	molluscs	111
34	AP00179	33	Human neutrophil peptide-4 (Alpha def)	Human	97
35	AP00641	33	Pardaxin 1	Fish	96
36	AP00771	23	Magainin 1	Frog	90
37	AP00742	42	Chicken AvBD6 (Beta def)	Chicken	88
38	AP00144	23	Magainin 2	Frog	85
39	AP00846	43	Mundticin KS (Bacteriocin)	Bacteria	66

**Table 3:** ClusPro ranking of docked complex based on cluster size (member), where peptides with (*∗*) represent the experimentally validated against MERS-CoV and are considered as positive controls.

Rank	Peptide	Length	Definition	Species	Representative	Member
1	AP00166	25	Pleurocidin	Fish	Center	134
2	AP00641	33	Pardaxin 1	Fish	Center	134
3	AP00144	23	Magainin 2	Frog	Center	117
4	AP00771	23	Magainin 1	Frog	Center	117
5	AP01644	30	Brevinin-2-RN1	Frog	Center	117
6	AP00764	24	Dermaseptin-S9	Frog	Center	110
7	AP02571	31	Cycloviolacin VY1 (cyclotides)	Plant	Center	110
8	AP00275	31	Circulin B (cyclotides)	Plant	Center	107
9	AP01022	31	Cycloviolin A (cyclotides)	Plant	Center	107
10	AP01061	31	Circulin D (cyclotides)	Plant	Center	107
11	AP00549	40	Plectasin (fungal defensin)	Fungus	Center	101
12	AP00729	29	Kalata B1 (cyclotides)	Plant	Center	101
13	AP00730	31	Kalata B8 (cyclotides)	Plant	Center	101
14	AP01065	31	Cycloviolacin 014 (cyclotides)	Plant	Center	101
15	AP01788	43	Myticin C	molluscs	Center	97
16	AP01356	46	Cp-thionin II	Plant	Center	93
17	AP00742	42	Chicken AvBD6 (Beta def)	Chicken	Center	87
18	AP02148	50	Apl-AvBD16 (Beta def)	Bird	Center	87
19	AP00846	43	Mundticin KS (Bacteriocin)	Bacteria	Center	83
20	AP00532	20	Lunatusin	Plant	Center	78
21	P9*∗*	30	Mouse Beta-defensin	Mouse	Center	68
22	AP00692	30	HFIAP-3 (Hagfish cathelicidin)	Fish	Center	67
23	AP00036	38	Bovine Beta-defensin 1	Bovine	Center	66
24	AP00074	24	Brevinin-1	Frog	Center	66
25	AP00744	41	AvBD-5, chicken avian beta defensin)	Chicken	Center	66
26	AP02663	23	Piscidins	Fish	Center	66
27	AP00179	33	Human neutrophil peptide-4 (Alpha def)	Human	Center	57
28	AP00174	31	Guinea pig neutrophil cationic peptide 1	Guinea pig	Center	49
29	AP02733	55	Piscidin	Fish	Center	43
30	AP00160	28	Dermaseptin-S4	Frog	Center	40
31	AP00180	32	Human defensin 5 (alpha defensin)	Human	Center	40
32	AP00222	32	RatNP-1 (rat alpha defensin,)	Rat	Center	40
33	AP00223	32	RatNP-2 (rat alpha defensin)	Rat	Center	40
34	HR2P*∗*	36	HR2 region of MERS-CoV	Synthetic	Center	39
35	AP00340	25	Chrysophsin-2	Fish	Center	38
36	AP00181	32	Human defensin 6	Human	Center	37
37	AP00260	22	Maculatin 1.1	Frog	Center	37
38	AP02830	43	ccBD (Channel Catfish beta def)	Fish	Center	33
39	AP00225	31	RatNP-4 (rat defensin)	Rat	Center	31

**Table 4:** ClusPro ranking of docked complex based on energy scores, where peptides with (*∗*) represent the experimentally validated against MERS-CoV and are considered as positive controls.

Rank	Peptide	Length	Definition	Species	Representative	Energy
1	AP00260	22	Maculatin 1.1	Frog	Center	-1692.0
2	AP02733	55	Piscidin	Fish	Center	-1581.2
3	AP00179	33	Human neutrophil peptide-4 (Alpha def)	Human	Center	-1498.5
4	AP00340	25	Chrysophsin-2	Fish	Center	-1488.6
5	AP00181	32	Human defensin 6	Human	Center	-1399.5
6	AP00180	32	Human defensin 5 (alpha defensin)	Human	Center	-1340.8
7	AP00222	32	RatNP-1 (rat alpha defensin,)	Rat	Center	-1340.8
8	AP00223	32	RatNP-2 (rat alpha defensin)	Rat	Center	-1340.8
9	AP00764	24	Dermaseptin-S9	Frog	Center	-1338.8
10	AP00742	42	Chicken AvBD6 (Beta def)	Chicken	Center	-1264.8
11	AP02148	50	Apl-AvBD16 (Beta def)	Bird	Center	-1264.8
12	HR2P*∗*	36	HR2 region of MERS-CoV	Synthetic	Center	-1256.9
13	AP00174	31	Guinea pig neutrophil cationic peptide 1	Guinea pig	Center	-1223.0
14	AP01788	43	Myticin C	molluscs	Center	-1202.7
15	AP02830	43	ccBD (Channel Catfish beta def)	Fish	Center	-1184.3
16	AP00074	24	Brevinin-1	Frog	Center	-1184.2
17	AP02663	23	Piscidins	Fish	Center	-1184.2
18	AP01356	46	Cp-thionin II	Plant	Center	-1139.1
19	AP00166	25	Pleurocidin	Fish	Center	-1137.1
20	AP00225	31	RatNP-4 (rat defensin)	Rat	Center	-1103.8
21	AP00036	38	Bovine Beta-defensin 1	Bovine	Center	-1103.7
22	AP00744	41	AvBD-5, chicken avian beta defensin)	Chicken	Center	-1103.7
23	AP00160	28	Dermaseptin-S4	Frog	Center	-1097.0
24	AP00641	33	Pardaxin 1	Fish	Center	-1050.1
25	AP00549	40	Plectasin (fungal defensin)	Fungus	Center	-994.2
26	AP00275	31	Circulin B (cyclotides)	Plant	Center	-993.9
27	AP01022	31	Cycloviolin A (cyclotides)	Plant	Center	-993.9
28	AP01061	31	Circulin D (cyclotides)	Plant	Center	-993.9
29	AP00846	43	Mundticin KS (Bacteriocin)	Bacteria	Center	-937.1
30	P9*∗*	30	Mouse Beta-defensin	Mouse	Center	-925.5
31	AP00532	20	Lunatusin	Plant	Center	-921.9
32	AP02571	31	Cycloviolacin VY1 (cyclotides)	Plant	Center	-897.7
33	AP00144	23	Magainin 2	Frog	Center	-868.2
34	AP00771	23	Magainin 1	Frog	Center	-868.2
35	AP01644	30	Brevinin-2-RN1	Frog	Center	-868.2
36	AP00692	30	HFIAP-3 (Hagfish cathelicidin)	Fish	Center	-821.3
37	AP00729	29	Kalata B1 (cyclotides)	Plant	Center	-805.8
38	AP00730	31	Kalata B8 (cyclotides)	Plant	Center	-805.8
39	AP01065	31	Cycloviolacin 014 (cyclotides)	Plant	Center	-805.8

**Table 5:** Binding mode of each peptide-protein complex using Protein Interaction Calculator (PIC) server.

APD3 ID	Hydrophobic interactions	Main chain Hydrogen bond interactions	Side chain Hydrogen bond interactions	Ionic interactions	Aromatic-aromatic interactions	Aromatic-sulphur interactions
P9	**Tyr64**, **Ile69**, Tyr809,** Ala920**, **Tyr928**, **Val929**, Tyr932, Ala1037	Asn812, Ser919, Asp922, Asn1042, Asn812, Ser1038	Tyr932	Glu1039	**Tyr928, Tyr64 **	Cys925

AP00549	Ala1049, Pro59, **Tyr64**, **Tyr928**, **Val929**, Ala930,** Ala920**, **Ile69**, Tyr71	Ala1049, Gly61	Gln60, Gln1056, Cys925	Arg1057, Arg62, Asp922	**Tyr928, Tyr71 **	**- **

AP00225	*Val790, Tyr1142*, Phe764, *Leu731*, Ile768, *Pro1143, Pro767, Val770*	Pro730	Thr791, Gln1119, Tyr1141, Leu729, Asn765, Leu731, His1146, Asn765, His766	Gln792, Ser734	Glu1017	Tyr1142

AP00180	Ala1007, Val790, *Leu731, Pro767*, Ile768, *Tyr1142*	Gly789, Pro730	Gln1119, Tyr1142, Leu731, Leu729, Pro730, Asn765 Aln1007	Glu1017, Asp740	-	Cys1142

AP00744	Leu1200, *Pro767, Val1168*, Ile1180, *Leu780*, Phe778, *Pro1143*, Val983, Ile985	Ala1206	Cys1164, Val770, Tyr1153, Ser781	Asp771	Tyr1142	-

HR2P	Tyr1153, Ile1165, *Val1168*, Ile1180, Val1181, *Leu780, Val770, Pro767, Pro1143, Tyr1142, Val790, Leu729,* Pro730, *Leu731*	-	Asn1169	His1146, His766	-	-

## Data Availability

The data used to support the findings of this study are available from the corresponding author upon request.

## Supplementary Materials

Supplementary Materials Supplementary Table 1 shows the model evaluation of modelled AMPs using Rampage Server.
