# Supplementary material for: Peptide-Protein Interaction Studies of Antimicrobial Peptides Targeting Middle East Respiratory Syndrome Coronavirus Spike Protein: An In Silico Approach
Source: Adv Bioinformatics. 2019 Jul 1;2019:6815105. doi: 10.1155/2019/6815105 (PMC6634063; doi:10.1155/2019/6815105)
Supplement: Supplementary Materials — Supplementary Table 1 shows the model evaluation of modelled AMPs using Rampage Server. [file 6815105.f1.pdf]

Table 1: Validation of modelled AMPs using Rampage servers

**Peptide**	**Rampage server**
(1) AP00074	Number of residues in favoured region (~98.0% expected) : 20 (90.9%)
	Number of residues in allowed region (~2.0% expected) : 1 (4.5%)
	Number of residues in outlier region : 1 (4.5%)
(2) AP00166	Number of residues in favoured region (~98.0% expected) : 23 (100.0%)
	Number of residues in allowed region (~2.0% expected) : 0 (0.0%)
	Number of residues in outlier region : 0 (0.0%)
(3) AP00275	Number of residues in favoured region (~98.0% expected) : 23 (79.3%)
	Number of residues in allowed region (~2.0% expected) : 4 (13.8%)
	Number of residues in outlier region : 2 (6.9%)
(4) AP00181	Number of residues in favoured region (~98.0% expected) : 25 (83.3%)
	Number of residues in allowed region (~2.0% expected) : 4 (13.3%)
	Number of residues in outlier region : 1 (3.3%)
(5) AP00340	Number of residues in favoured region (~98.0% expected) : 23 (100.0%)
	Number of residues in allowed region (~2.0% expected) : 0 (0.0%)
	Number of residues in outlier region : 0 (0.0%)
(6) AP00549	Number of residues in favoured region (~98.0% expected) : 31 (81.6%)
	Number of residues in allowed region (~2.0% expected) : 3 (7.9%)
	Number of residues in outlier region : 4 (10.5%)
(7) AP00729	Number of residues in favoured region (~98.0% expected) : 24 (88.9%)
	Number of residues in allowed region (~2.0% expected) : 3 (11.1%)
	Number of residues in outlier region : 0 (0.0%)

(8) AP00730	Number of residues in favoured region (~98.0% expected) : 22 (75.9%)
	Number of residues in allowed region (~2.0% expected) : 5 (17.2%)
	Number of residues in outlier region : 2 (6.9%)
(9) AP02571	Number of residues in favoured region (~98.0% expected) : 22 (75.9%)
	Number of residues in allowed region (~2.0% expected) : 5 (17.2%)
	Number of residues in outlier region : 2 (6.9%)
(10) AP02733	Number of residues in favoured region (~98.0% expected) : 36 (67.9%)
	Number of residues in allowed region (~2.0% expected) : 9 (17.0%)
	Number of residues in outlier region : 8 (15.1%)
(11) AP00180	Number of residues in favoured region (~98.0% expected) : 25 (83.3%)
	Number of residues in allowed region (~2.0% expected) : 3 (10.0%)
	Number of residues in outlier region : 2 (6.7%)
(12) AP00225	Number of residues in favoured region (~98.0% expected) : 23 (79.3%)
	Number of residues in allowed region (~2.0% expected) : 2 (6.9%)
	Number of residues in outlier region : 4 (13.8%)
(13) AP00744	Number of residues in favoured region (~98.0% expected) : 34 (87.2%)
	Number of residues in allowed region (~2.0% expected) : 3 (7.7%)
	Number of residues in outlier region : 2 (5.1%)
